# The Role of Potassium Channel Activation in Celecoxib-Induced Analgesic Action

**DOI:** 10.1371/journal.pone.0054797

**Published:** 2013-01-24

**Authors:** Yao Mi, Xuan Zhang, Fan Zhang, Jinlong Qi, Haixia Gao, Dongyang Huang, Li Li, Hailin Zhang, Xiaona Du

**Affiliations:** 1 The Key Laboratory of Neural and Vascular Biology, Ministry of Education, Department of Pharmacology, Shijiazhuang, China; 2 Department of New Drug Development, Hebei Medical University, Shijiazhuang, China; Virginia Commonwealth University, United States of America

## Abstract

**Background and Purpose:**

Celecoxib (CXB) is a widely prescribed COX-2 inhibitor used clinically to treat pain and inflammation. Recently, COX-2 independent mechanisms have been described to be the targets of CXB. For instance, ion channels such as the voltage-gated sodium channel, L-type calcium channel, Kv2.1, Kv1.5, Kv4.3 and HERG potassium channel were all reported to be inhibited by CXB. Our recent study revealed that CXB is a potent activator of Kv7/M channels. M currents expressed in dorsal root ganglia play an important role in nociception. Our study was aimed at establishing the role of COX-2 independent M current activation in the analgesic action of CXB.

**Methods and Results:**

We compared the effects of CXB and its two structural analogues, unmethylated CXB (UMC) and 2,5-dimethyl-CXB (DMC), on Kv7/M currents and pain behavior in animal models. UMC is a more potent inhibitor of COX-2 than CXB while DMC has no COX-2 inhibiting activity. We found that CXB, UMC and DMC concentration-dependently activated Kv7.2/7.3 channels expressed in HEK293 cells and the M-type current in dorsal root ganglia neurons, negatively shifted I–V curve of Kv7.2/7.3 channels, with a potency and efficiency inverse to their COX-2 inhibitory potential. Furthermore, CXB, UMC and DMC greatly reduced inflammatory pain behavior induced by bradykinin, mechanical pain behavior induced by stimulation with von Frey filaments and thermal pain behavior in the Hargreaves test. CXB and DMC also significantly attenuated hyperalgesia in chronic constriction injury neuropathic pain.

**Conclusion:**

CXB, DMC and UMC are openers of Kv7/M K^+^ channels with effects independent of COX-2 inhibition. The analgesic effects of CXBs on pain behaviors, especially those of DMC, suggest that activation of Kv7/M K^+^ channels may play an important role in the analgesic action of CXB. This study strengthens the notion that Kv7/M K^+^ channels are a potential target for pain treatment.

## Introduction

In clinical practice, non-steroid anti-inflammatory drugs (NSAIDs) are the most frequently used pain relief drugs. It is believed that NSAIDs mainly relieve pain by suppressing the activity of cyclooxygenase (COX) [1], [2], which, as it is a rate-limiting enzyme in the conversion of arachidonic acid to prostaglandin (PG), reduces PG generation. PGs, particularly PGE2, are well-known mediators of inflammation and pain [3], [4]. Three COX isozymes have been characterized so far, COX-1-3. COX-1 and COX-2 are of particular interest because they are the major targets of NSAIDs.

COX-1 is a ubiquitous constitutive form of the enzyme that is involved in the regulation of various physiological processes such as platelet aggregation, and gastrointestinal tract and kidney homeostasis. COX-2 is an inducible isozyme mainly observed during pathological processes such as inflammation and cancer [5]. In this regard, COX-2 inhibitors were expected to be safer due to the lack of gastrointestinal and other NSAID-related side-effects associated with COX-1 inhibition. However, the safety of COX-2 inhibitors came into question after they were approved for clinical use when rofecoxib (Vioxx) and some other COX-2-specific inhibitors were shown to significantly increase the risk of cardiovascular events and were thus voluntarily withdrawn from the market [6], [7]. Presently celecoxib (CXB) is the only COX-2 inhibitor still in clinical use.

Recently, COX-2-independent mechanisms have been described to be the targets of CXB. For instance, several non-COX-2 components of the cell, such as sarcoplasmic/endoplasmic reticulum (ER) calcium ATPase (SERCA) [8] and 3-phosphoinositide-dependent protein kinase-1 (PDK1) [9], [10], have been identified and proposed as candidates for mediating the COX-2-independent anti-tumor effects of CXB.

Certain ion channels were also recently described as additional targets of CXB. For example, the voltage-gated sodium channel in rat retinal neurons [11] and dorsal root ganglia (DRG) neurons [12], [13], the L-type calcium channel in rat pheochromocytoma (PC12) cells [14] and A7r5 rat aortic smooth muscle cells [15], Kv2.1 channels expressed in HEK293 cells [16], cardiac Kv1.5, Kv4.3 and Kv7.1 channels in guinea pig cardiomyocytes [17] and human eag-related gene (HERG) potassium channels [18] were all reported to be inhibited by CXB. In contrast, Kv7.5 currents in rat A7r5 aortic smooth muscle cells can be acutely augmented by CXB [15]. Our recent study on CXB modulation of the Kv7 family revealed that CXB is a potent activator of Kv7.2-4 but an inhibitor of Kv7.1 [19]. Our study also suggested that the effects of CXB on Kv7 channels depend on its direct binding to the channel rather than its COX-2 inhibition [19].

The strong modulation of Kv7.2/7.3 by CXB found in our previous work led us to think that activation of K^+^ channels may also contribute to the analgesic action of CXB. It is well established that Kv7.2 and Kv7.3 (coded by KCNQ2 and KCNQ3, respectively) constitute the molecular basis of the neuronal M-type potassium channel [20], [21]. M currents are voltage- and time-dependent, low threshold, slow activating, slow deactivating and non-inactivating outward K^+^ currents [22]. The low threshold (−60 mV) activation of M currents makes the current the key factor in determining the neuronal resting membrane potential and excitability. M channels are found in the sympathetic ganglia, DRG, hippocampus and other central nervous system regions [23], [24], [25]. Recent work suggest that M currents expressed in DRG neurons play important roles in nociception and activation of the M channels by the M channel activator retigabine (RTG) inhibits response to the intrapaw application of carrageenan [26] and bradykinin (BK) [27] in rat behavioral studies. Furthermore, the M channel blocker XE991 evokes spontaneous pain in rats [27], [28]. In our earlier study, we attributed BK-induced acute pain to the inhibition of M currents and the activation of Ca^2+^-activated Cl^−^ currents in DRG neurons [27].

In light of the findings mentioned above, we propose that, in addition to reducing the generation of PGs by inhibiting COX-2, activation of M currents in DRG neurons may also be involved in the NSAID analgesic action of CXB. In the present study, we compared the effects of CXB and its two structural analogues, unmethylated CXB (UMC) and 2,5-dimethyl-CXB (DMC), on Kv7/M currents and pain behavior in animal models. As UMC is a more potent inhibitor of COX-2 than CXB and DMC has no COX-2-inhibiting activity, the role of COX-2-independent M current activation in the analgesic action of CXB can be assessed by comparing the effects of these three CXB analogues.

## Results

### CXBs Significantly Increase the Kv7.2/7.3 Current Expressed in HEK293 Cells

**Figure 1:**
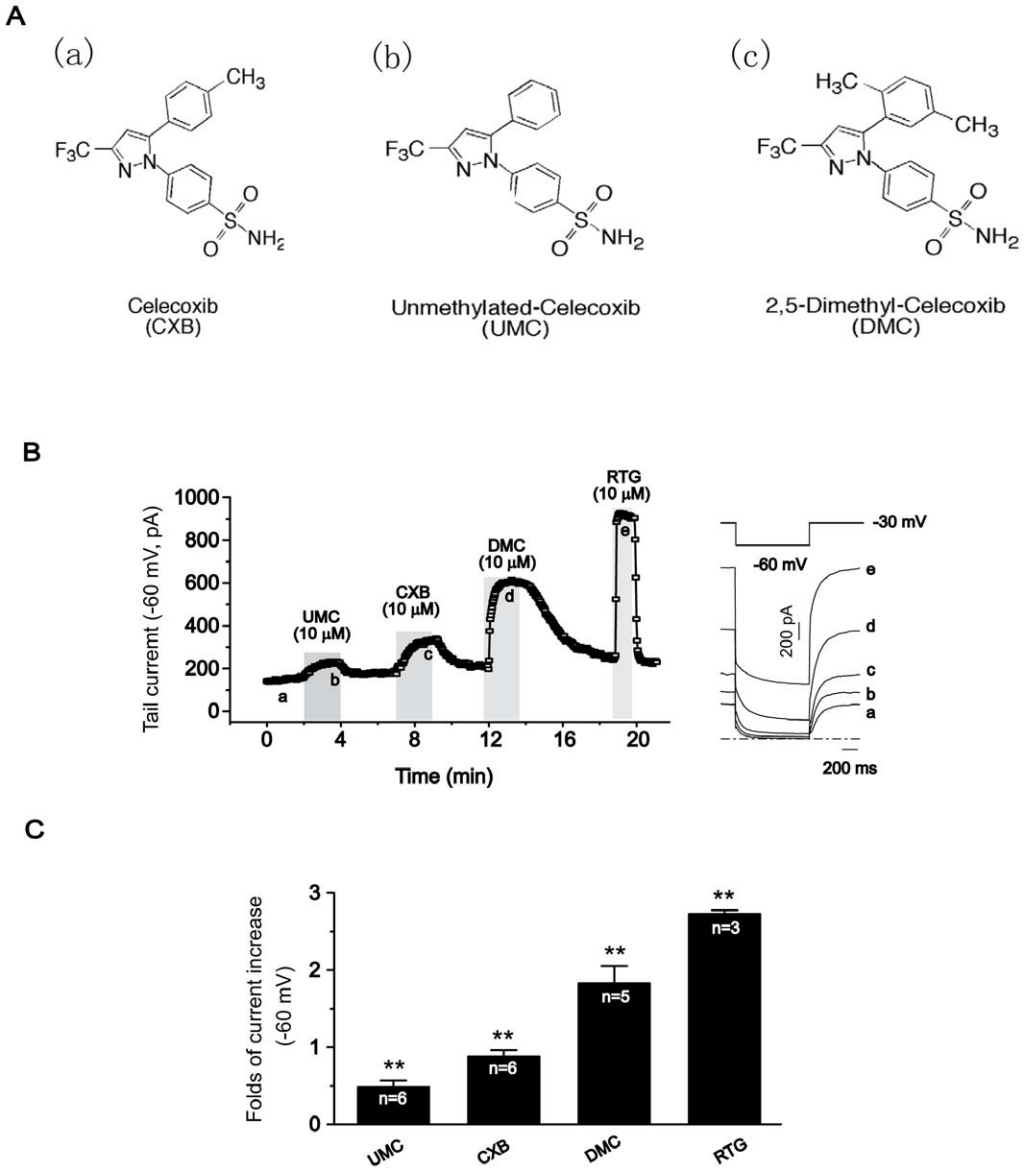
Effects of CXB analogues on Kv7.2/7.3 expressed in HEK293 cells. A, Structures of CXB analogues. (a), Celecoxib (CXB); (b), unmethylated-celecoxib (UM-celecoxib, UMC); (c), 2, 5-Dimethyl-celecoxib (DM-celecoxib, DMC) B, The time course for the effects of CXB, UMC and DMC on the tail currents (−60 mV) of Kv7.2/7.3 recorded using the protocol shown at the top of the right panel. The representative current traces under different conditions of the treatments were shown at the right. The dotted line indicates the zero current level. C, Summarized data for the effects of CXB analogues on Kv7.2/7.3 channel. **P < 0.01, compared with the control current before application of CXB analgoues or retigabine (RTG). n = 3–6.

UMC and DMC are structural analogues of CXB (Figure 1A). With regards to the inhibitory capability of COX-2, UMC is a more potent inhibitor of COX-2 than CXB while DMC lacks COX-2 inhibition. As such, UMC and DMC are valuable tools for pinpointing the COX-dependent and -independent effects of CXB. In our previous work, we proved that both CXB and DMC are activators of Kv7.2/7.3 channels [19]. In the first part of this study, we further characterized the effects of CXB, DMC and UMC on Kv7.2/7.3 channels expressed in HEK293 cells.

The Kv7.2/7.3 currents were recorded using the protocol shown in the right panel of Figure 1B and measured as the deactivating tail currents at −60 mV relaxed from the activated currents at −20 mV. Figure 1B shows the time course of the deactivating tail currents and the representative activating and deactivating currents of Kv7.2/7.3, before and after external application of 10 µM UMC, CXB and DMC. It is clear that all three drugs reversibly enhanced the Kv7.2/7.3 current at this concentration: UMC, CXB and DMC increased the Kv7.2/7.3 tail currents recorded at −60 mV by 50.2 ± 7.1%, 87.4 ± 7.5% and 181.8 ± 21.9%, respectively. RTG, an established activator of Kv7.2/7.3, increased the Kv7.2/7.3 current by 272.1 ± 5.4% (Fig. 1C).

### CXBs Concentration-dependently Activate Kv7.2/7.3 Channel Expressed in HEK293 Cells

**Figure 2:**
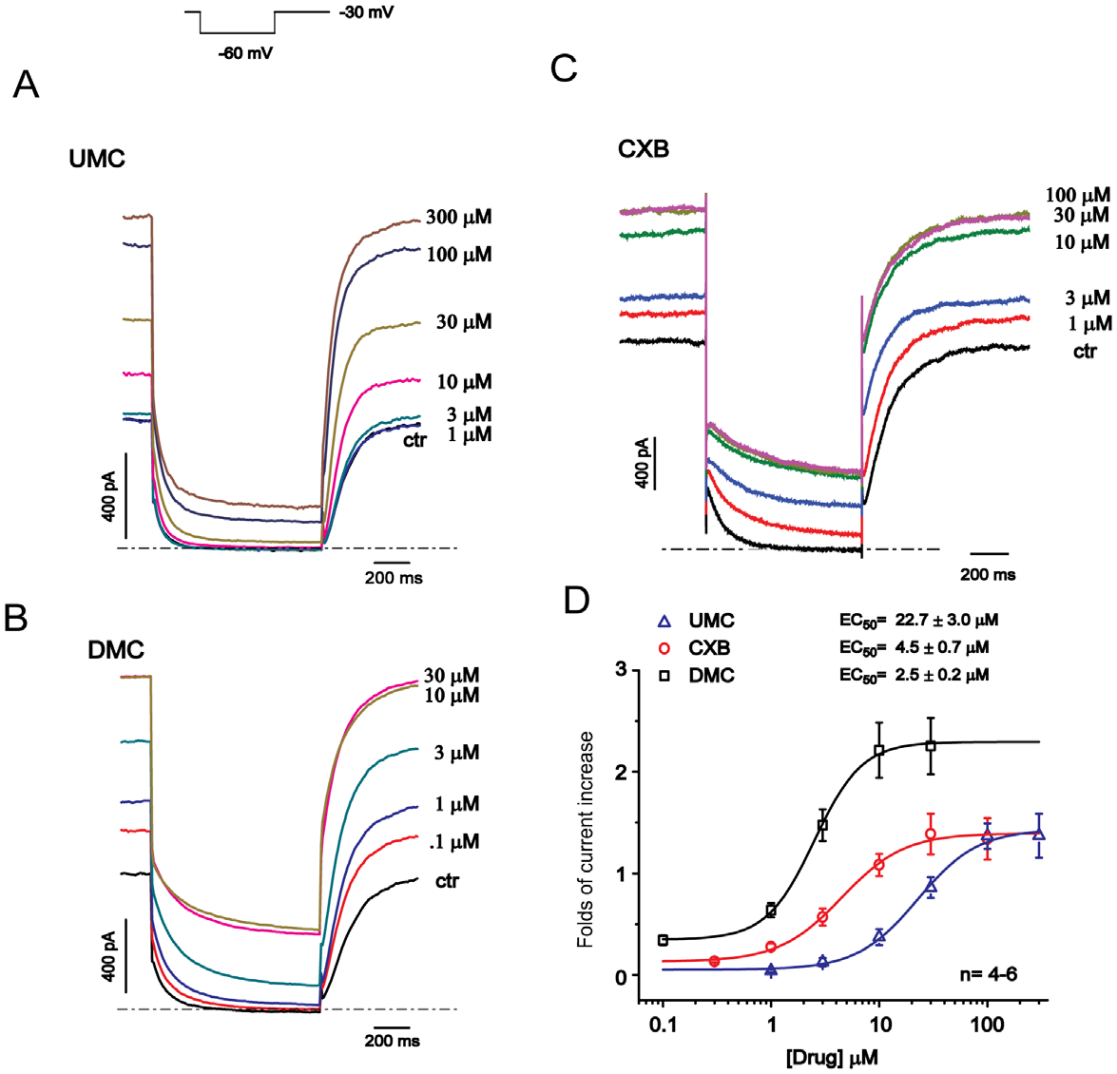
Concentration-dependent effects of CXB analogues on Kv7.2/7.3 expressed in HEK293 cells. The currents were recorded using the protocol shown at the left top. A–C, Representative traces of Kv7.2/7.3 current responding to the different concentrations of UMC, DMC and CXB were shown. The dotted line indicates the zero current level. D, The concentration-response relationships for activation of Kv7.2/7.3 currents by CXB analogues were fitted with the logistic function. Folds of currents increased at – 30 mV were plotted against drug concentrations. The EC_50_ value is 22.7 ± 3.0 µM for UMC, 4.5 ± 0.7 µM for CXB, and 2.5 ± 0.2 µM for DMC. n = 4–6.

Next, we examined the concentration-response relationships for the UMC, CXB and DMC activation of Kv7.2/7.3 channels. Figures 2A–C show the representative traces of Kv7.2/7.3 currents before and after application of a concentration series of the three drugs. All compounds concentration-dependently enhanced the Kv7.2/7.3 currents recorded at both −60 mV and −20 mV (Fig. 2D). The maximal percentage increase of Kv7.2/7.3 currents induced by UMC, CXB and DMC administration were 140.1 ± 14.2%, 143.3 ± 21.4% and 227.9 ± 24.3%, respectively, while the EC_50_ for the Kv7.2/7.3 current activation were 22.7 ± 3.0 µM, 4.5 ± 0.7 µM, and 2.5 ± 0.2 µM for UMC, CXB and DMC, respectively. Thus, the order of both efficacy and potency of these CXBs were UMC < CXB < DMC, which is opposite to the order of their COX-2 inhibition activity (UMC > CXB > DMC). These results strongly suggest that the modulation of Kv7.2/7.3 currents by CXB analogues does not depend on their COX-inhibitory activity.

### CXBs Negatively Shift I–V Curve of Kv7.2/7.3 Channel Expressed in HEK293 Cells

**Figure 3:**
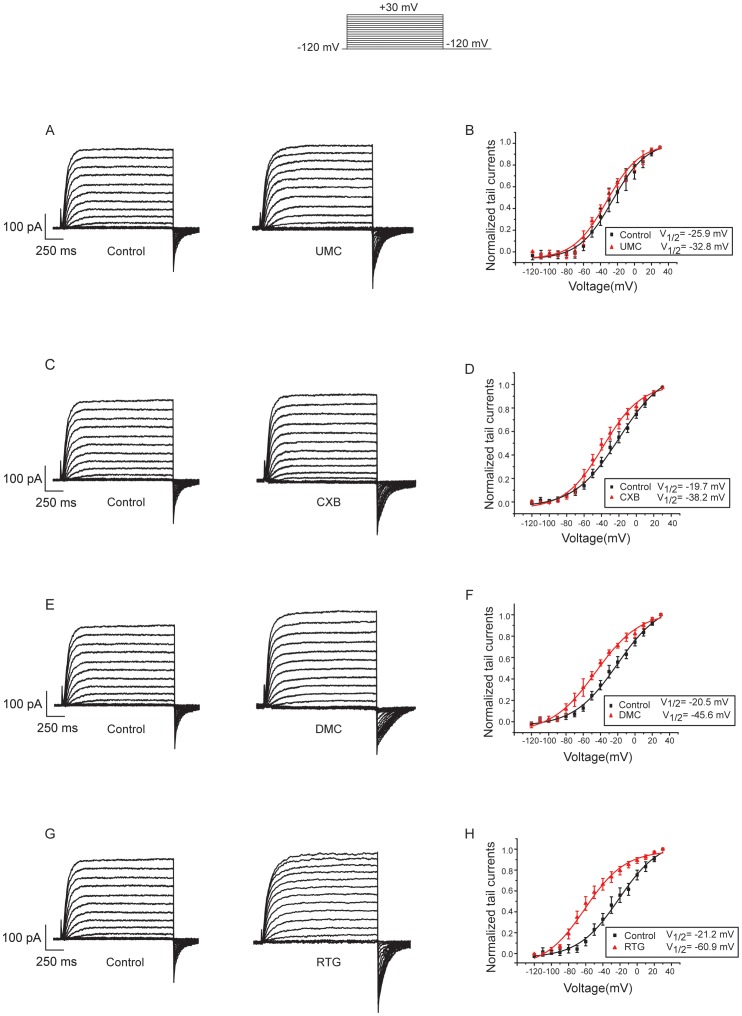
Effects of CXB analogues on voltage dependent activation of Kv7.2/7.3 expressed in HEK293 cells. The currents were recorded using the voltage protocol shown at the top of the Figure. A–D, Current traces of Kv7.2/7.3 before and after applying 100 µM CXB analogues and RTG. E–H, Voltage dependent activation curves were plotted from the tail currents recorded at – 120 mV against the preceding voltage step. The data were fitted with a Boltzmann function described in the Method. The voltages for the half maximal activation of Kv7.2/7.3 (V_1/2_) were shown. CXB analogues and retigabine (RTG) negatively shifted the voltage dependent activation of Kv7.2/7.3. n = 4–6.

The protocol shown at the top of Figure 3 was used to examine the effects of CXB analogues on voltage-dependent activation of Kv7.2/7.3 expressed in HEK293 cells. Figure 3A–D showed the current traces of Kv7.2/7.3 current before and after applying 100 µM CXB, DMC, UMC and RTG. Both the steady state currents at the end of each voltage step and tail currents of Kv7.2/7.3 were variously increased by CXBs and RTG at 100 µM. Figure 3E–H showed I–V curves plotted from the tail currents recorded at –120 mV following the preceding voltage step. The maximum tail current of each group was used to normalize all the tail currents in the same group. The V_1/2_ from the fitting of Boltzmann function (see Methods) showed that CXB, DMC and UMC shifted voltage dependent activation of Kv7.2/7.3 to more negative potentials to different degrees (Fig. 3E–G), indicating Kv7.2/7.3 channel would be activated in more negative potentials in the presence of CXBs. The order of effects for CXBs on I–V curve shifting were DMC > CXB> UMC, similar to the order of effects on current augmentation induced by CXBs. RTG showed the greatest effect on shifting the voltage dependent activation curve of Kv7.2/7.3 (Fig. 3H ).

### CXBs Increase the M-type K^+^ Currents from DRG Neurons

Our previous work showed that CXB and DMC not only activated Kv7.2/7.3 currents expressed in cell lines but also activated the native M-type K^+^ currents from rat super cervical ganglia (SCG) neurons [19]. In addition, our recent work [27] suggests that the M-type K^+^ currents in DRG neurons are an important modulator of nociception. In this study, we explored the effects of UMC, CXB and DMC on M-type K^+^ currents from rat DRG neurons.

**Figure 4:**
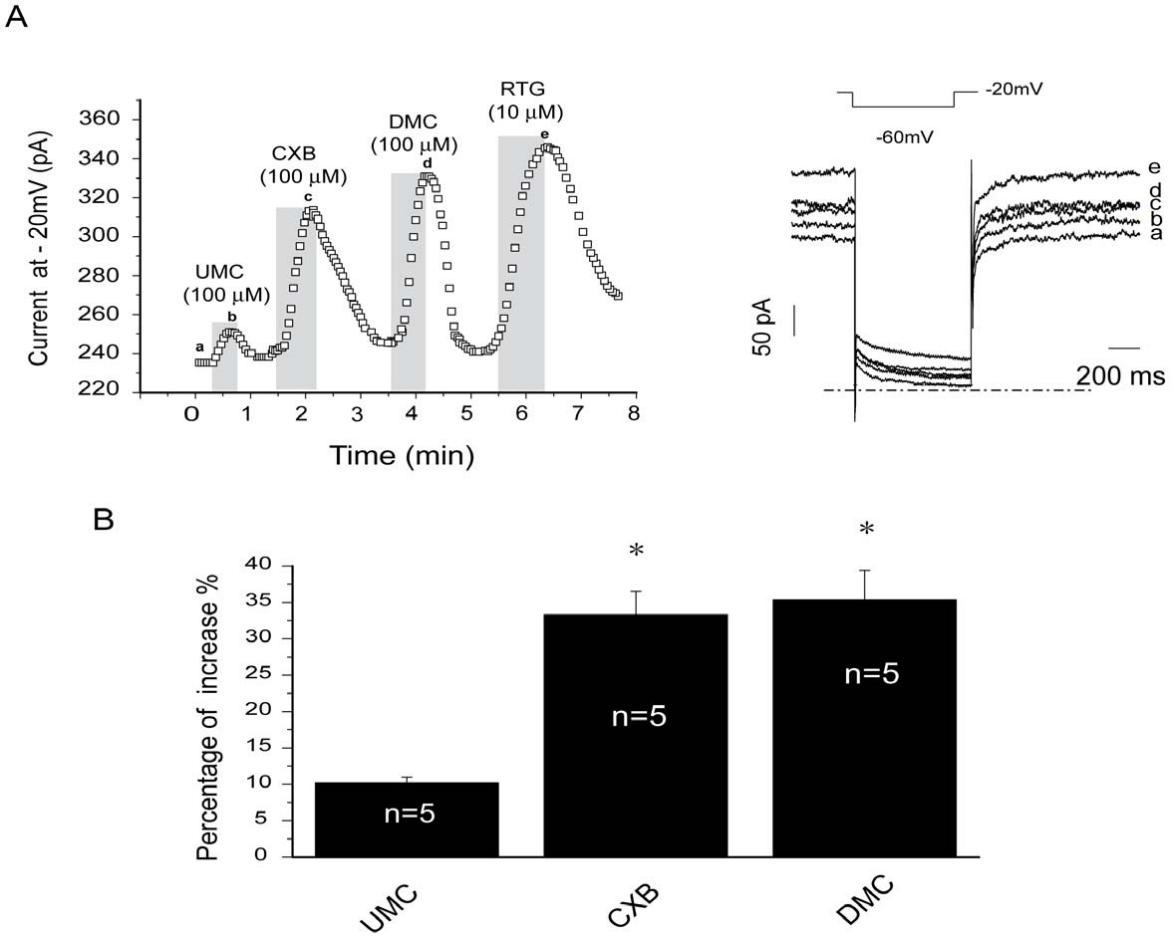
Effects of CXB analogues on M-type K^+^ currents from rat DRG neurons. A, The time course for the effects of CXB, UMC, DMC and RTG on the M-type K^+^ currents recorded using the protocol shown at the top of the right panel. The representative current traces under different conditions of the treatments were shown at the right. The dotted line indicates the zero current level. B, Summarized data for the effects of CXB analogues on M currents recorded at −20 mV. *P<0.05, compared with the control currents before application of celecoxib analogues. n = 5.

M-type K^+^ currents were recorded from small-diameter DRG neurons by amphotericin B perforated patch clamp using the voltage protocol depicted in Figure 4A (right panel). All three drugs increased M-type K^+^ currents from DRG neurons (Fig. 4A and 4B), by 10.1 ± 0.8%, 33.3 ± 3.6% and 36.3 ± 3.2% at 100 µM (Fig. 4B); at a lower concentration (10 µM), the effect was reduced but with the same order of potency (data not shown). RTG at 10 µM significantly increased M-type K^+^ currents (Fig. 4A). Thus, similar to the effects on expressed Kv7.2/Kv7.3 currents, these three drugs increased the M-type K^+^ currents in DRG neurons with the same order of potency: DMC > CXB > UMC.

### CXBs Attenuate the Inflammatory Pain Induced by BK

**Figure 5:**
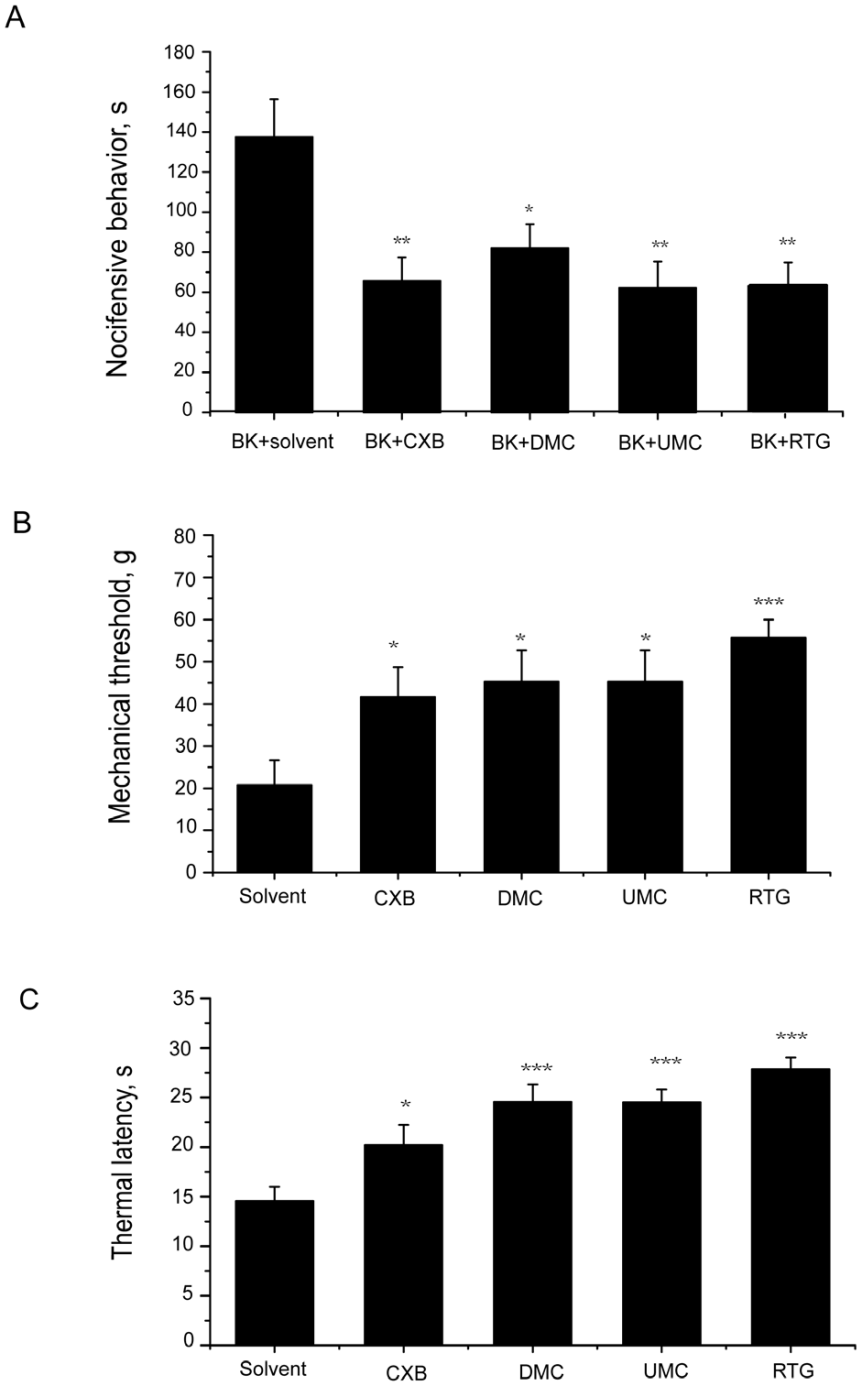
CXB analogues attenuate nocifensive behavior induced by BK, thermal and mechanical stimuli. A, Effects of CXB, UMC) DMC and RTG on BK-induced nocifensive behavior. 100 µM CXB, UMC, DMC or RTG was injected 5 min before the injection of BK (200 µM) plus solvent or the corresponding compounds into hind paw in volume of 50 µl in each case. The second injection was done into the same site. The time of animals spent licking, biting, lifting and flitching during 30 min after injection were recorded and shown. n = 10 for each group. *P <0.05, **P <0.01, compared with the BK plus solvent group. B, Effects of CXB, UMC, DMC and RTG on mechanical-induced nocifensive behavior. 100 µM CXB, UMC, DMC, RTG or solvent was were injected into rat hind paw in volume of 50 µl. 8 min later, paw withdrawal thresholds (g) were measured using calibrated Von Frey filaments applied to the plantar surface of the injected paw. n = 10. *P <0.05, ***P <0.001, compared with the solvent group. C, Effects of CXB, UMC, DMC and RTG on thermal-induced nocifensive behavior. 100 µM CXB, UMC, DMC, RTG or solvent was were injected into rat hind paw in volume of 50 µl. 8 min later, the injected hind paw was subjected to radiant heat from underneath the glass floor with a high-intensity lamp bulb, and paw withdrawal latency was measured and presented. n = 10. *P <0.05, ***P <0.001, compared with the solvent group.

The results presented so far suggest that Kv7/M channels in nociceptors may be a target for the analgesic action of CXB. We thus tested the effects of CXBs on the nociceptive behaviors of rat pain models, first using BK-induced inflammatory pain. For this, we evaluated the nocifensive response (time spent licking, biting and flinching the affected paw) following the hind paw injection of 50 µl of saline containing the relevant compounds. Intraplantar injection of BK (200 µM) into the hind paw produced strong nocifensive behavior (quantified within the first 30 min after injection; BK, 137.5 ± 18.9 s, Fig. 5A), which was not observed in rats injected with solvent (0.5% DMSO in saline, data not shown). Co-application of DMC, CXB or UMC (all at 100 µM) with BK (following a pre-application of the CXBs; see Methods for details) all greatly reduced BK-induced pain behavior. As demonstrated in Figure 5A, UMC, CXB and DMC reduced the BK-induced nocifensive time to 62.1 ± 13.2 s, 65.6 ± 11.8 s and 82.0 ± 12.1 s, respectively. The solvent control for CXBs did not affect BK-induced nociceptive effects (data not shown). Furthermore, consistent with our previous study, 100 µM RTG also reduced the BK-induced nocifensive time to 60.3 ± 10.3 s. These results suggest that activation of Kv7/M currents by CXBs could alleviate the acute inflammatory pain induced by BK.

### CXBs Antagonize the Nocifensive Response to Mechanical Stimuli

We used von Frey filaments to test for the withdrawal threshold to mechanical stimuli applied to the hind paw of rats. The solvent-containing saline control or relevant CXBs (50 µl) were injected into the plantar of the rat hind paw and the response to the mechanical stimuli was measured 8 min later. As demonstrated in Figure 5B, all of these CXBs significantly increased the thresholds for nocifensive withdrawal of the hind paw in response to the mechanical stimuli compared with the solvent control. The nocifensive withdrawal threshold in the solvent group was 20.7 ± 5.9 g while DMC, CXB and UMC (all at 100 µM) increased the thresholds to 45.2 ± 7.2 g, 41.6 ± 7.1 g and 55.7 ± 4.3 g, respectively. RTG (100 µM) also increased the threshold to 56.1 ± 7.4 g.

### CXBs Antagonize Thermal Pain Behavior

We used the Hargreaves test [29], in which the paw is heated by a radiant heat source, to study the latency of paw withdrawal from thermal stimuli. Thermal nocifensive behavior was studied 8 min after the intraplantar injection of 50 µl of saline containing solvent or the CXBs. As demonstrated in Figure 5C, compared with the solvent control, all of these CXBs increased the time latencies for thermal nociceptive behavior at a 100 µM concentration. The time latency in the solvent group was 14.4 ± 1.4 s while DMC and CXB and UMC increased the time latencies to 24.5 ± 1.8 s, 20.1 ± 2.0 s and 24.5 ± 1.2 s, respectively. RTG (100 µM) increased the time latency to 27.8 ± 1.2 s.

### The Effects of CXBs on Neuropathic Pain in the Rat Chronic Constriction Injury Model

**Figure 6:**
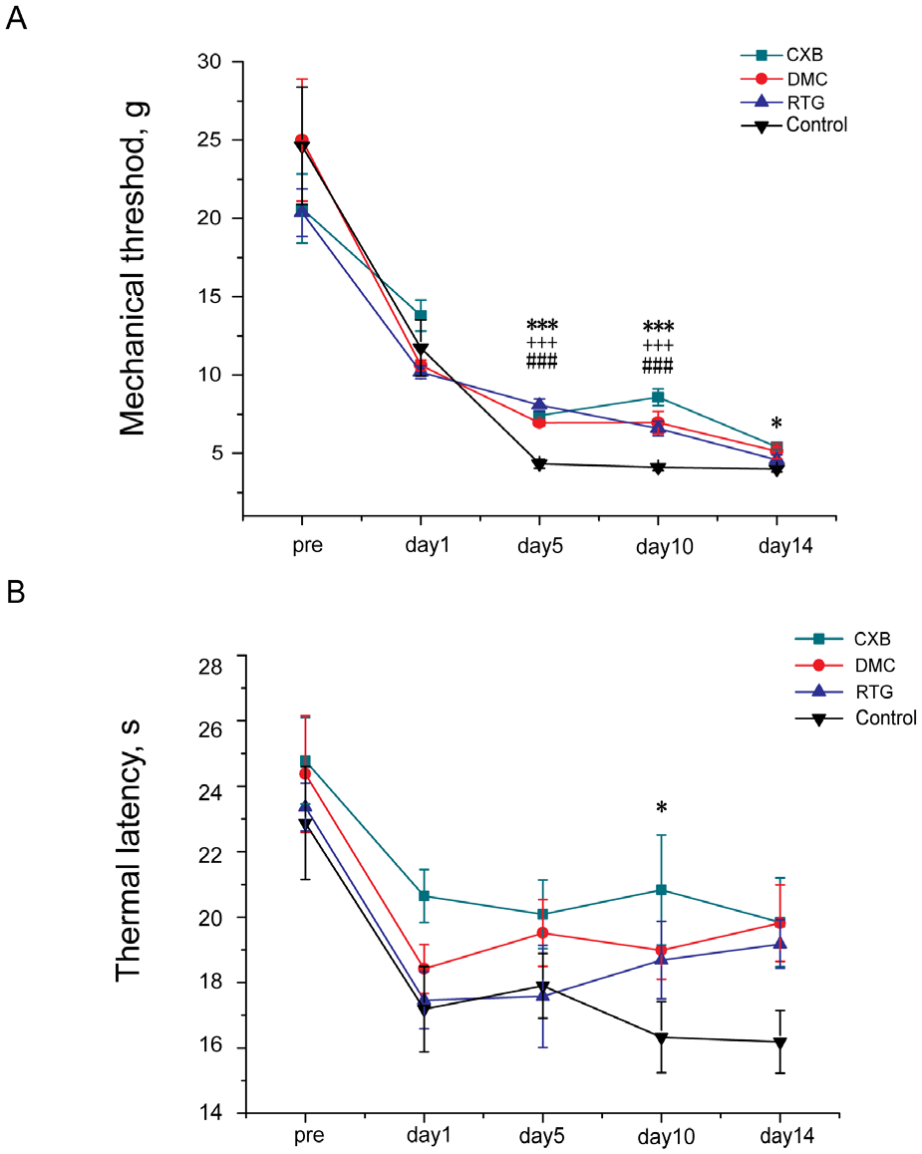
Effects of CXB analogues on neuropathic pain in chronic constriction injury (CCI) rats. CCI rats were given CXB, DMC, RTG (30 mg/kg/day) or solvent (intragastric (*i.g*) administration twice a day) from 1∼14 days after surgery. A, Effects of CXB, DMC and RTG on rat pain behavior responding to the mechanical stimuli. Mechanical thresholds (g) were measured using calibrated Von Frey filaments applied to the plantar surface of the ipsilateral hind paw before operation and 1day, 5days, 10 days and 14 days after the surgery. *P <0.05, ***P <0.001, CXB group compared with the solvent group., ^###^ *P* <0.001, RTG group compared with the solvent group, ^+++^ *P* <0.001, DMC group compared with the solvent group. B, Effects of CXB, DMC and RTG on rat pain behavior responding to the thermal stimuli. Thermal withdrawal latencies of the ipsilateral hind paw were measured using radiant heat from underneath the glass floor with a high-intensity lamp bulb before operation and 1 day, 5 days, 10 days and 14 days after surgery. *P <0.05, CXB group compared with the solvent group.

The neuropathic pain model of chronic constriction injury (CCI) to the sciatic nerve was used in this part of the study. Nocifensive response changes to mechanical or thermal stimuli after surgery were monitored using von Frey filaments or the Hargreaves test as discussed above. As shown in Figure 6, basic mechanical withdrawal thresholds and thermal pain latencies showed no differences between the control, CXB, DMC and RTG groups before the operation, which was in the range of 21–25 g, while the basic thermal pain thresholds in each group, which was in the range of 23–24 s, also did not show any differences.

On the first day after surgery, the mechanical pain threshold and thermal pain threshold were significantly decreased. From the first day after the surgery, the rats were divided into four groups with each group receiving intragastric administration of either CXB, DMC or RTG (all in a dose of 30 mg/kg/day) or solvent in a volume of 1 ml, twice a day (for details see Methods).

On the 5^th^ day after surgery, the mechanical pain threshold in the solvent group was further reduced to 4.3 ± 0.3 g, and all three drug treatments significantly increased the threshold compared with the solvent control (CXB, 7.4 ± 0.2 g; DMC, 7.0 ± 0.2 g; RTG, 8.1 ± 0.4 g). On the other hand, drug treatment did not affect the reduced thermal pain thresholds (solvent control, 18.4 ± 1.0 s; CXB, 20.1 ± 1.1 s; DMC, 19.3 ±1.0 s; RTG, 17.2 ± 1.6 s).

On the 10^th^ day after surgery, the mechanical pain thresholds in the drug groups were significantly increased compared with the solvent group (solvent, 4.1 ± 0.2 g; CXB, 8.5 ± 0.5 g; DMC, 7.0 ± 0.7 g; RTG, 6.6 ± 0.5 g). For the thermal pain threshold, only CXB treatment significantly increased the threshold (solvent, 16.3 ± 1.1 s; CXB, 20.8 ± 1.7 s).

On the 14^th^ day after surgery, only CXB treatment significantly increased mechanical threthold compared with the solvent treatment (for the mechanical pain threshold: solvent, 4.0 ± 0.2 g; CXB 5.4 ± 0.2 g). For the thermal pain threshold, none of the treatments significantly affected the thermal latencies: control, 16.1 ± 0.9 s; CXB, 19.8 ± 1.3 s; DMC, 19.8 ± 1.2 s; RTG, 19.1 ± 0.7 s).

## Discussion

Growing evidence suggests that functional Kv7/M channels are expressed in peripheral sensory neurons and fibers and that their activity strongly contributes to fiber excitability [24], [30], [31]. Activation of Kv7/M channels by an opener like RTG inhibits animal pain behavior [26]–[27] while inhibition of the Kv7/M channel with a blocker like XE991 evokes spontaneous pain [27], [28]. Thus, it is logical to hypothesize that a drug (such as CXB) with the ability to activate Kv7/M channels is able to antagonize nociception. To prove this hypothesis, CXB and its two analogues with different levels of COX-2 inhibition were used in this study. One of the analogues, DMC, lacks the ability to inhibit COX-2 [32] and is often used for studying the COX/PGE2-independent effects of CXB. The other CXB analogue, UMC, has a higher potency of COX-2 inhibition than CXB [33]. By comparing these three CXBs for their effects on Kv7/M channel activation and on animal pain behavior, we should be able to clarify whether activation of K^+^ conductance plays a role in the analgesic action of CXB. We found that all three CXB analogues concentration-dependently activate Kv7/M channels, with both potency and efficacy of the stimulatory effects inversely related to their COX-2 inhibitory activity: DMC showed the greatest effect while UMC showed the weakest effect in activating Kv7/M channels. Furthermore, CXB analogues showed similar order of potency on negatively shifting I–V curve of Kv7 channel. These results support our previous study showing that CXB modulates Kv7/M channel in a COX-2 independent manner [19] and that CXB makes Kv7/M channel more easily to be activated at negative potentials. Similar activation effect trends of CXBs for native M-type K^+^ currents from DRG neurons were also observed, except that higher drug concentrations were required in DRG neurons.

Characterization of the effects of CXB analogues on Kv7/M currents lays a solid foundation for proving our hypothesis that activation of K^+^ currents contribute to the analgesic actions of CXB. Thus, if all three CXB analogues with different potencies of COX-2 inhibition relieve pain similarly, it would strongly suggest that K^+^ channel activation is a target for the analgesic action of CXB. In this regard, DMC is particularly valuable, given that DMC, like CXB, activates Kv7/M channels but lacks COX-2 inhibitory activity. We found that CXB, UMC and DMC could attenuate inflammatory pain induced by BK. CXB and UMC, both inhibitors of COX-2, seemed more effective than DMC (Fig. 5A), which could be due to the fact that PGE2 is an important mediator during the inflammation process. Moreover, all of these drugs were also able to antagonize both mechanical pain and thermal pain. The effectiveness of DMC in all these pain models indicates that Kv7/M current modulation by CXBs indeed contribute to the alleviation of inflammatory, mechanical and thermal pain.

Neuropathic pain is characterized by hyperalgesia, allodynia and spontaneous pain and is notoriously difficult to treat. The analgesic effects of NSAIDs on neuropathic pain are still being debated. Some studies suggest that injured nerve-derived COX-2/PGE2 contributes to the maintenance of neuropathic pain [34], [35]. In the present study, both CXB and DMC were found to be able to relieve neuropathic pain to some extent in CCI rats (Fig. 6). For neuropathic pain manifested by the heightened responses to the mechanical stimuli, the effects of CXB and DMC were similar, and the analgesic effects started at one day after the surgery. For the thermal pain in the CCI rats, CXB seemed more effective than DMC, which suggests that COX-2/PGE2 is possibly involved in the development of the neuropathic pain. On the other hand, the effectiveness of both DMC and RTG indicate that Kv7/M current activation could be an effective approach for treating neuropathic pain. Our results are consistent with observations that CXB can reduce neuropathic pain, especially mechanical allodynia in a brachial plexus avulsion model [36], [37].

In summary, our results suggest that, apart from inhibiting COX-2, activation of Kv7/M K^+^ currents may also contribute to the analgesic action of CXB. To our knowledge, this is the first experimental evidence that ascribes a non-COX-inhibitory mechanism to the analgesic action of a NSAID. With recent evidence that many NSAIDs can affect the functions of non-COX proteins, our results imply a need for further evaluation of NSAID effects that are independent of COX inhibition.

## Methods

### Materials

CXB, UMC and DMC were synthesized in the Department of New Drug Development, School of Pharmacy, Hebei Medical University. The stock solutions for CXB (100 mM), DMC (100 mM) and RTG (100 mM) were dissolved in DMSO and stored at −20°C. The other chemicals were all purchased from Sigma (St. Louis, MO, USA).

### Ethics Statement

The use of animals in this studied was approved by the Animal Care and Ethical Committee of Hebei Medical University (Shijiazhuang, China) under the International Association for the Study of Pain (IASP) guidelines for animal use. All surgery was performed under sodium pentobarbital anesthesia and all efforts were made to minimize suffering.

### HEK293 Cells Culture and Transfection

The HEK293 cell line was purchased from American Type Culture Collection (ATCC, maryland, USA). HEK293 cells were cultured in Dulbecco’s modified Eagle’s medium (DMEM) supplemented with 10% fetal bovine serum and antibiotics in a humidified incubator at 37°C (5% CO_2_). The cells were seeded on glass coverslips in a 24-multiwell plate and transfected when 60–70% confluence was reached. For transfection of six wells of cells, a mixture of 3 µg Kv7.2 and Kv7.3 in pcDNA3 (1.5 µg for each), pEGFP-N1 cDNAs and 3 µl Lipofectamine 2000 reagent (Invitrogen, USA) were prepared in 1.2 ml DMEM and incubated for 20 min according to the manufacturer’s instructions. The mixture was then applied to the cell culture wells and incubated for 4–6 h. Recordings were made 24 h after transfection and the cells were used within 48 h.

### Rat DRG Cell Culture

The DRG were extracted from all spinal levels of 21-day-old male Sprague Dawley rats, and the neurons were dissociated as previously described [27]. Briefly, the rats were anesthetized with an intraperitoneal injection of sodium pentobarbital (10–20 mg/kg) and then sacrificed. The ganglia were cut into pieces, transferred into a collagenase solution (1 mg/ml) and incubated for 30 min at 37°C. The ganglia were then placed into a trypsin solution (2.5 mg/ml) for 20 min at 37°C. The digested fragments were then rinsed three times with 2 ml DMEM with 10% fetal bovine serum, centrifuged and dissociated by trituration. The ganglia were plated onto glass coverslips pre-coated with poly-D-lysine and incubated at 37°C. After the neurons had attached to the coverslips, fresh cell culture medium was added to 1 ml. Neurons were used 3–5 days after isolation. The diameters of the DRG neurons were measured using a calibrated micrometer mounted in the eyepiece of the microscope.

### Electrophysiology

Patch electrodes were pulled from borosilicate glass and fire-polished to a final resistance of 1–2 MΩ when filled with internal solution. An axon 700B (Axon Instruments, USA) patch clamp amplifier was used for voltage clamp experiments. All recordings were performed using the amphotericin B (250 µg/ml, Sigma, St. Louis, MO, USA) perforated patch technique. The internal pipette solution contained (in mM): 150 KCl, 5 MgCl_2_, 10 HEPES, pH 7.4. The external solution contained (in mM): 160 NaCl, 2.5 KCl, 5 CaCl_2_, 1 MgCl_2_, 10 HEPES, 8 glucose, pH 7.4. A low-profile perfusion chamber fed by gravity perfusion system was used for solution exchange (2 ml/min, bath exchange time of ∼15 s).

### Behavioral Studies

Male Sprague Dawley rats (180–220 g) were randomly grouped and allowed to acclimatize for at least 20 min to the environment prior to the experiment. All experimenters were blinded to the treatment allocation and were only unblinded once the study had finished.

#### BK-induced acute spontaneous pain

The right hind paw of the animal received an intraplantar injection (50 µl) of BK (200 µM, 10 nM/site) and the nocifensive response (licking, biting, lifting and flinching) were recorded using a video camera for 30 min. To study the effects of drugs on BK-induced nociceptive behavior, animals were pre-injected with CXB analogues or RTG. After 5 min, BK and the drug were co-injected into the same site of the hind paw. Control animals were injected with solvent (0.5% DMSO in saline) instead of the tested drugs. All drugs were diluted in saline from stock solution and applied at a volume of 50 µl at a concentration of 100 µM.

#### Mechanical pain

Mechanical withdrawal thresholds were measured using calibrated von Frey filaments (a set of monofilaments made from nylon filaments of varying diameter) (North Coast Medical, Morgan Hill, CA, USA) applied to the plantar surface of the paw. Testing was initiated with an Evaluator Size 5.07 (10 g). If the animal withdrew the paw, the next weaker hair was applied. In the case of no withdrawal, the next stronger hair was applied. The cut-off was Evaluator Size 6.10 (100 g).

#### Thermal pain

To test for thermal hyperalgesia, radiant heat was applied to the plantar surface of a hind paw from underneath a glass floor using a ray of light from a high-intensity lamp bulb. The paw withdrawal latency was recorded automatically when the paw was withdrawn from the light (Taimeng Technology, Chengdu, China).

#### Neuropathic pain

CCI was used as a model of neuropathic pain. Animals were randomly divided into 4 groups that received either CXB, DMC, RTG or solvent treatment. After one day of environment acclimatization, basic mechanical and thermal withdrawals were assessed. The surgeries were performed one day later. The rats were anesthetized with an intraperitoneal injection of sodium pentobarbital (10–20 mg/kg). The left hind leg was shaved and cleaned using 70% ethanol. The sciatic nerve was exposed by blunt preparation of connective tissue at the mid-thigh level, proximal to the sciatic trifurcation. Four non-absorbable sterile surgical sutures (0.1 mm) were loosely tied around the sciatic nerve, 1–1.5 mm apart. The skin was sutured and the animal was transferred to a recovery cage. CCI rats received the vehicle (0.5% sodium carboxymethyl cellulose) or CXB, DMC or RTG treatment (30 mg/kg/day) by intragastric administration twice a day in a volume of 1 ml from 1 day to 14 days after the surgery. Mechanical and thermal withdrawals were tested at 1, 5, 10 and 14 days after surgery using the methods described above.

### Data Analysis and Statistics

The concentration-response curve was fitted by logistic equation: y = A_2_+(A_1_−A_2_)/(1+(x/x_0_)^p^), where *x* is the drug concentration, and *p* is the Hill coefficient. The current activation curves were generated by plotting the normalized tail current amplitudes against the step potentials and were fitted with a Boltzmann equation: y = A/{1+exp[(*V*_h_−*V*_m_)/*k*]}, where *A* is the amplitude of relationship, *V_h_* is the voltage for half-maximal activation, *V*_m_ is the test potential, and *k* is the slope factor of the curve. All data are reported as the mean ± standard error of the mean (SEM). Differences between groups were assessed by a Student’s *t*-test or one-way analysis of variance (ANOVA) followed by Bonferroni’s post-hoc test. The differences were considered significant if P ≤0.05.
